# Prevalence of Nonpathogenic *Avibacterium paragallinarum* in Naïve-Healthy Layer Flocks Across Multiple States in the United States

**DOI:** 10.1155/tbed/9994679

**Published:** 2025-05-18

**Authors:** Mostafa M. S. Shelkamy, Amro Hashish, Mariela E. Srednik, Maria Chaves, Nubia R. Macedo, Eman Gadu, Stephan Schmitz-Esser, Qijing Zhang, Chong Wang, Yuko Sato, Mohamed El-Gazzar

**Affiliations:** ^1^Department of Veterinary Diagnostic and Production Animal Medicine, College of Veterinary Medicine, Iowa State University, Ames 50011, Iowa, USA; ^2^Department of Avian and Rabbit Medicine, Faculty of Veterinary Medicine, Suez Canal University, Ismailia 41522, Egypt; ^3^National Laboratory for Veterinary Quality Control on Poultry Production, Animal Health Research Institute, Agriculture Research Center, Giza 12618, Egypt; ^4^Department of Veterinary Sciences, University of Wyoming, Laramie 82070, Wyoming, USA; ^5^Department of Avian and Rabbit Diseases, Faculty of Veterinary Medicine, Mansoura University, Mansoura 35516, Egypt; ^6^Department of Animal Science, Iowa State University, Ames 50011, Iowa, USA; ^7^Department of Veterinary Microbiology and Preventive Medicine, College of Veterinary Medicine, Iowa State University, 1800 Christensen Drive, Ames 50011, Iowa, USA; ^8^Department of Statistics, Iowa State University, Ames 50011, Iowa, USA

**Keywords:** *Avibacterium paragallinarum* (AP), infectious coryza (IC), layer chickens, nonpathogenic *Avibacterium paragallinarum* (npAP), pilot surveillance study, quantitative real-time PCR (qPCR)

## Abstract

Infectious coryza (IC) caused by *Avibacterium paragallinarum* (AP) is an emerging infectious respiratory disease in the commercial chicken layer industry in the Midwestern US states. Outbreak investigations around positive index cases led to the discovery of nonpathogenic AP (npAP), which caused quantitative real-time PCR (qPCR) positive results in naïve-healthy layer (NHL) flocks. Therefore, the reliability of positive qPCR as confirmed diagnosis of IC became questionable and the poultry industry was deprived from an essential diagnostic tool in the face of an actively spreading outbreak. However, the prevalence of npAP in NHL flocks and the magnitude of this diagnostic challenge remained unclear. This pilot surveillance study aims to provide an initial estimate of npAP prevalence in the US commercial layer industry. Two differential qPCR assays were recently developed to differentiate pathogenic AP (pAP) and npAP. A total of 710 oropharyngeal (OP) swab pools (5 swabs/pool) were collected from 80 NHL sites across 13 US states and tested using qPCR assay targeting the *recN* gene as a screening test. Two hundred thirty-one out of 710 total pools were positive for npAP (32.5%) representing 28 positive sites out of the 80 total sites (35%). All positive qPCR samples from NHL flocks were confirmed to be due npAP. The differential qPCR was capable of confirming 85.71% of the npAP cases, while the remaining cases required further isolation and whole-genome sequencing (WGS). In conclusion, this pilot study indicates that the prevalence of npAP in NHL flocks in the United States is above 30%. Therefore, in flocks with no clinical signs, qPCR assays cannot be relied upon for IC diagnostic confirmation. Currently, isolation combined with WGS is the only diagnostic tool capable of completely differentiating between these two AP populations, which indicates the immediate need for improvements in the available diagnostic assays.

## 1. Introduction

Infectious coryza (IC) is a respiratory contagious disease of chickens, associated with infraorbital sinus inflammation with facial swelling and sharp decrease in feed and water intake, which consequently lead to a significant decline in egg production up to 40% in laying hens and growth retardation in broilers [1]. *Avibacterium paragallinarum* (AP), the causative agent of IC, is a primary pathogen [2], implying that its presence in naïve-healthy susceptible chickens should result in visible illness. Although the clinical picture of IC is obvious and a presumptive diagnosis can be easily reached, confirmed laboratory diagnosis is needed before making any of the costly decisions necessary for disease control. Disease control measures and in turn confirmed diagnosis become even more crucial in the case of an emerging infectious disease outbreaks. Disease control decisions are significantly costlier in case of a multiage layer complex (MAC), which highlights the need for a confirmed diagnosis in these cases. Confirmed diagnosis of IC can be achieved by bacterial isolation, which is challenging due to the fastidious nature of AP that grows slowly and is often overgrown with other commensals, including other *Avibacterium* species [2, 3]. Therefore, using quantitative real-time PCR (qPCR) became much more popular as a confirmatory diagnostic assay. As a result, multiple qPCR assays were developed and are currently available for the confirmatory diagnosis of IC [4–7].

While the clinical signs are drastic and obvious, mortality induced by IC is often low [8], unless complicated by other pathogens or additional stress factors [9, 10]. After the initial acute clinical phase of IC, surviving individuals and flocks become chronic carriers [11]. For this reason, IC has been historically endemic in the Southern United States, from California in the west coast to Georgia and Florida in the east. However, this disease has rarely been documented in the northern latitudes of North America [12]. This epidemiological narrative of IC being “a-warm-weather-disease” was changed with the recent emergence of IC in a large outbreak in commercial poultry in Pennsylvania in 2018–2019 [13, 14]. Shortly after the Pennsylvania outbreak, neighboring states, Delaware and Maryland, reported IC cases [5]. In 2020, an IC outbreak spread west from Pennsylvania, affecting one large MAC in Ohio, resulting in severe clinical signs and significant drops in egg production. In late spring of 2023, the outbreak moved further west and a large number of IC cases were reported from western Ohio and eastern Indiana [15]. In September of 2023, the IC outbreak has extended further west to affect one MAC in eastern Iowa (personal communication). Genetic information, at the level of both partial *HMTp210* gene and at the whole-genome sequencing (WGS) as well as verified epidemiological links confirm that Pennsylvania outbreak is indeed the source of the subsequent spread of AP to the eastern shore and into midwestern states (unpublished data). These cases are the first-ever reported in commercial poultry this far north in the United States. Therefore, accurate diagnosis, appropriate surveillance assays and monitoring programs are needed to curtail the spread of this disease into such areas where it is not endemic yet.

As part of the effort by the veterinarian who diagnosed the initial index case in Ohio in 2020, to define the scale of the outbreak and to attempt to contain the spread of IC, several MAC around the initial index case were tested for AP on pooled choanal swabs (five birds per pool/six pools per flock/site) using the qPCR assay available at that time [4] and later tested by the qPCR assay targeting the *recN* gene that was developed in 2021 [5].

The outbreak investigation efforts during 2020 and 2021 included naïve-healthy layer (NHL) flocks with no history of previous IC exposure and no clinical signs. However, multiple of these NHL flocks included in the outbreak investigation yielded positive qPCR results, with *C*_T_ values ranging from 25.6 to 34.1. These results were surprising due to the absence of any apparent IC clinical signs such as swollen sinuses or decreased feed intake, water consumption, or egg production. These characteristic IC clinical signs were never reported before in the history of these layer flocks, not at the time of sampling, and never developed at any time after the time of collecting these samples and the positive PCR results (personal communication). This observation was in a stark contrast with the severe clinical signs observed in the index case. These qPCR positive results with lack of clinical signs could not be explained as recovered chronic carrier status due to the lack of any previous history of the disease or vaccination in these flocks and in the entirety of this midwestern states of the United States. Hereafter, in this manuscript, these healthy layer flocks with no history of IC disease and have never developed any IC clinical signs will be referred to as NHL flocks.

**Figure 1:**
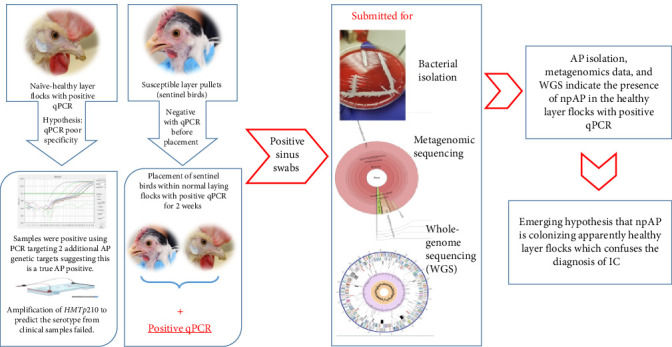
Illustration of the initial discovery of npAP in naïve-healthy layer flocks during the IC outbreak investigation during 2020 and 2021.

To further investigate these results, sentinel birds were introduced into these NHL flocks (Figure 1). Thirty naïve 10-week-old sentinel birds were placed in two of the qPCR positive caged NHL flocks. The sentinel birds tested negative by qPCR before placement and 15 days later, the sentinel birds were delivered to Iowa State University–Veterinary Diagnostic Laboratory (ISU-VDL), where they were necropsied and choanal swabs pools (five birds per pool) were tested using the currently available qPCRs [4, 5]. Almost all pools tested positive with *C*_T_ values ranging between 24.9 and 31.3. Despite testing positive on qPCR, the sentinel birds remained completely healthy, with no clinical signs and no gross lesions. This experiment was repeated once more, with the only difference being that the sentinel birds were placed for 21 days, with very similar results.

Interestingly, pure AP isolates were acquired out of both the sentinel birds and the original layer flocks. The acquired AP isolates were genetically characterized and the genomic analysis revealed that they exhibited genetic divergence from the classical pathogenic AP (pAP) strains in some genetic markers like *HMTp210* gene unique insertions and the lack of capsular polysaccarhide locus (including *hctA*), while still considered within the species of AP based on the genomic average nucleotide identity (ANI) [16]. Due to the lack of clinical disease in both NHL flocks and sentinel birds colonized by these AP isolates, these isolates were dubbed non-pAP (npAP). Based on the genetic differences, two targets were selected to develop and validate two differential qPCR assays that are capable of differentiating between pAP and npAP [17].

The presence of such npAP population in the commercial poultry industry has already resulted in a significant confusion of IC diagnosis and undermined confidence in the qPCR results, which is the most popular confirmatory tests for the disease. In turn, this led to uncertainty regarding the disease status in some flocks and hesitation in making economically costly decisions necessary for the prevention, control, and eradication of this emerging disease. However, the scope and scale of the problem remains unknown. It could be a localized phenomenon affecting only one or a few integrators or a widespread issue impacting big proportions of the layer industry. Due to our lack of knowledge about this newly discovered population of npAP, the purpose of this pilot study is to investigate the prevalence of npAP in NHL flocks throughout the US commercial layer industry and provide preliminary estimates on the magnitude of this diagnostic challenge.

## 2. Materials and Methods

### 2.1. Sample Size and Collection

#### 2.1.1. Sample Size at the Level of the US Layer Population (Layer Site Is the Epidemiological Unit)

This study is intended to be a pilot study to provide an initial estimate of the prevalence of npAP in naïve-healthy commercial layer population. In general, the goal of pilot studies is not to accurately estimate the true prevalence; therefore, power or sample size calculations are often not performed. In this study, the considered sample size, at the population level, is not for purpose of true prevalence estimation; it is rather to provide preliminary data that can be used for power calculations and sample size in future more comprehensive studies. Additionally, this data will provide an estimate of the magnitude of this diagnostic challenge. For pilot study sample size, different studies suggest numbers between 12 and 30 units [18, 19]. However, in our pilot study we aimed to include 100 units (layer sites). We aimed to collect samples from a minimum of 10 NHL sites from each of the top 10 egg-producing states [20]. However, we were unable to fully achieve this due to various challenges. Sample collection largely depended on our collaborators, field veterinarians in different states, and some were unable to gather samples from 10 sites per state. The endemic presence of IC in Pennsylvania made it nearly impossible to obtain samples from NHL flocks, as many flocks were previously exposed and are chronic carriers. Therefore, our collaborators in that state could not find sites meeting the inclusion criteria, leading to the exclusion of Pennsylvania from our study.

#### 2.1.2. Inclusion Criteria

Only commercial layer sites in production with no history of exposure to IC, and no previous or current clinical signs suggestive of IC were included in this study and referred to as NHL flocks. Our primary target for this study were NHL flocks located in the top 10 producing states; however, flocks from states that are not top egg producing states were included as well. Veterinarians expressed interest in determining whether npAP was present at their sites outside the top 10 producing states. Site selection was based on accessibility, availability, and permissions granted by farm owners and collaborating veterinarians.

#### 2.1.3. Sample Size Within the Epidemiological Unit

To detect a positive signal by qPCR, a sample size was estimated using the simple binomial model (https://epitools.ausvet.com.au/freecalctwo), assuming a minimum prevalence of npAP of 10% within the flock and perfect testing conditions, with 95% confidence [21, 22]. A sample size of 30 bird oropharyngeal (OP) swabs, grouped into six pools (five birds per pool), was estimated for each site. Each site was typically represented by a single house, from which all six pools were collected. However, in cases involving MACs and single-flock-multihouse (SFMH) systems, more than one house was often included. In these instances, six pools were usually collected from each house, with some exceptions due to submitter limitations, as shown in Table S1.

A total of 710 OP swab pools were collected in brain–heart infusion (BHI) broth from 3550 birds across 80-layer sites in 13 different US states. These states, in alphabetical order, were Arkansas, Georgia, Indiana, Iowa, Michigan, Minnesota, Missouri, Nebraska, North Carolina, Ohio, Oregon, Texas, and Washington. The sampling period spanned from December 2022 to March 2024. Nine out of the top 10 egg producing states were included in this study. The samples represented 40 MAC, 24 SFMH (consisting of two or more houses of the same ages, all-in-all-out sites), and 16 single-flock-single-house (SFSH) layer sites with only one house and one flock onsite (individual flocks, all-in-all-out sites). The age of the sampled birds ranged from 17 to 106 weeks (wks.). A detailed breakdown of sample distribution by state is shown in Table 1. All samples in this study were received from unvaccinated flocks with the exception of seven out of 80 sites (8.75%), which where flocks that had been previously vaccinated with inactivated IC vaccines. This vaccination was not expected to affect the qPCR results, as the flocks had no history of prior exposure to IC infection.

### 2.2. DNA Extraction and qPCR

The Kingfisher-Flex machine and MagMAX Pathogen RNA/DNA Kit (Thermo Fisher Scientific, Waltham, MA, USA) were used following the manufacturer's instructions for the extraction of nucleic acids from both OP swabs and clinical isolates. Nucleic acid extraction was performed using 100 µL of each sample/isolate, with elution into 90 µL of elution buffer. We spiked exogenous internal positive control (XIPC) at a concentration of 6250 copies per reaction and used the unpublished XIPC qPCR assay developed by ISU-VDL to monitor potential PCR inhibitors.

Samples were initially tested by qPCR assay targeting the *recN* gene [5] as the screening qPCR assay for the comprehensive identification of all AP (including both pAP and npAP). Positive samples were subsequently tested using the two recently developed differential qPCR assays described by Shelkamy et al. [17]. The primer and probe sequences used are shown in Table 2. The two newly developed differential qPCR assays were used to confirm that the positive signals detected in the NHL flocks by the *recN* screening qPCR were indeed due to the presence of npAP. Briefly, these qPCR assays consist of the np-*HMTp210* assay, which targets a specific and unique insertion in the *HMTp210* gene of npAP isolates, which is present only in npAP, and the *hctA* assay, which targets a capsular polysaccharide transporter gene (*hctA*) present only in pAP, as determined by comparative genomic analysis. Sites that are positive by the screening qPCR because of npAP are expected to be positive on the assay targeting the *HMTp210* gene unique insertion and negative on the assay targeting the *hctA* gene (positive–negative sites).

All qPCR assays used the same protocol and were applied using the Real-Time PCR System 7500 (Applied Biosystems, Carlsbad, CA, USA) in the standard 7500 run mode. A total reaction volume of 20 µL was applied, comprising TaqMan Fast Virus 1-step Master Mix (5 µL; Applied Biosystems, Carlsbad, CA, USA), primers (0.4 µmol final concentration), probe (0.2 µmol final concentration), XIPC primers (0.2 µmol final concentration), XIPC probe (0.075 µmol final concentration), 5 µL of DNA template, and PCR grade water up to the final volume. The amplification conditions were as follows: a cycle of 50°C for 5 min; then a denaturation cycle at 95°C for 20 s without optics; followed by 40 cycles of denaturation at 95°C for 15 s and annealing/extension at 60°C for 60 s with optics on.

Each test run incorporated a negative control (PCR-grade H_2_O) and a positive control (DNA extracted from npAP and pAP isolates confirmed by next generation WGS). Analysis of results was conducted using the SDS 1.5.1 software (Applied Biosystems, Carlsbad, CA, USA) with Manual *C*_T_ and Auto Baseline analysis settings. *C*_T_ values were determined by multiplying the delta Rn of the peak fluorescence by 0.05 [23]. Samples were considered AP positive at *recN C*_T_ ≤ 35.0 and XIPC *C*_T_ < 40.0 based on in-house validation testing.

### 2.3. Bacterial Isolation and Identification

Once a sampling site tested positive by the screening *recN* qPCR on the initial OP swabs pools, heads from freshly euthanized birds were submitted for bacterial isolation. For efficient isolation of AP, we used two new selective culture media that do not require nurse bacteria and can inhibit the growth of background commensal bacteria (unpublished). Following the standardized procedures for isolation, swabs from the infraorbital sinus were plated on MSNV and MSCV agar. These two media are composed of Mueller–Hinton agar, fetal bovine serum, nicotinamide adenine dinucleotide, and inhibitors (unpublished). After 48 h of incubation at 37°C with 5.2% of CO_2_, AP like colonies were restreaked. Subsequent confirmation of AP isolation was conducted using MALDI-TOF [24] and the qPCR assays mentioned in Section 2.2.

### 2.4. WGS and ANI Score

WGS of bacterial AP isolates was performed using Illumina MiSeq system (Illumina, USA) and MinIon Nanopore (Oxford Nanopore Technology (ONT), UK). This was done to differentiate pAP from npAP by confirming the presence or absence of the genetic targets utilized in the differential qPCR assays. In brief, the extracted DNA from 11 npAP isolates obtained during this study was used to prepare sequencing libraries with the Nextera XT DNA Library Prep Kit (Illumina, USA), producing 300 bp paired-end reads. For ONT sequencing, genomic DNA extraction was performed on the same 11 npAP isolates using the Genomic-tip 20/G kit (Qiagen, Germany). Library preparation was conducted using the Ligation Sequencing Kit (SQK-LSK109) and barcoded with the Native Barcoding Expansion (EXP-NBD114), following ONT instructions. JSpeciesWS service [25] was used to calculate the ANI score, utilizing the ESV-135 reference strain (NZ_CP050316.1) as a representative strain for AP species. The complete and whole genome sequences of the 11 npAP isolates are available in the GenBank under the following accessions: CP173231–CP173234, JBJBHF000000000 (positive–negative npAP), CP173229, CP173230, CP173236, JBJBHD000000000 (double-positive npAP), and CP173228, JBJBHC000000000 (double-negative npAP).

### 2.5. Statistical Analysis

SAS v9.4 was used for statistical analysis. Statistical difference in average *C*_T_ values and ages between included states was evaluated using the analysis of variance (ANOVA). The difference in the average age between the positive and negative flocks was compared using a *t*-test. The age comparison was based on site-level data, which could include different houses of varying ages on the same site. In such cases, we used the average age for the entire site. However, the house-level data was presented as detailed descriptive data to ensure precision and accuracy, as some sites showed positive results in certain houses, but negative in others.

## 3. Results

### 3.1. Real-Time PCR Results

**Figure 2:**
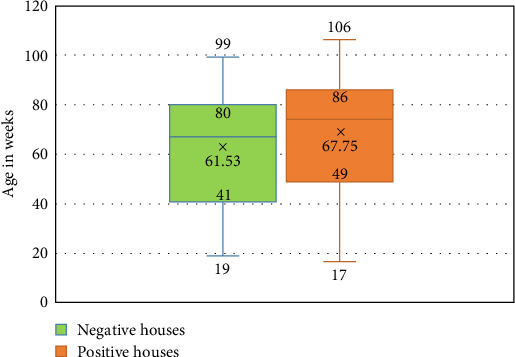
Distribution of age (in weeks) among the npAP negative and positive houses within the 13 US states included in the study. Each box and whisker plot shows the four quartiles and the average that is denoted by x.

In the US states analyzed in this study, eight out of 13 (61.53%) exhibited a positive AP signal in NHL flocks by the screening *recN* assay. Even if only one flock tested positive, the entire state was considered positive. These positive states are, in alphabetical order, Georgia, Indiana, Iowa, Michigan, Missouri, North Carolina, Ohio, and Texas. All the positive states are in the top 10 egg-producing states. We identified a total of 28 positive sites out of the 80 sites tested by the *recN* screening assay, comprising 23 MAC (*n* = 40), four SFMH (*n* = 24) layer sites, and one SFSH (*n* = 16) layer sites. This indicates that 35% of the tested NHL sites are positive by the screening qPCR assay. Regarding the *C*_T_ values among the positive sites, there was no significant differences (*p*=0.3860) between positive sites, with a *C*_T_ range from 21.54 to 34.94 and a *C*_T_ average of 29.41 ± 2.62. There was a significant difference (*p*=0.0039) in the age averages between positive and negative sites among all tested sites. The distribution of ages at the house level is shown in Figure 2, where the average age of 61.53 ± 21.7 wks. was reported in the negative and an average age of 67.75 ± 24.29 wks. was reported in the positive houses. Fifty percent of the negative houses ranged from 41–80 wks., while ranged from 49–86 wks. in the positive ones.

**Figure 3:**
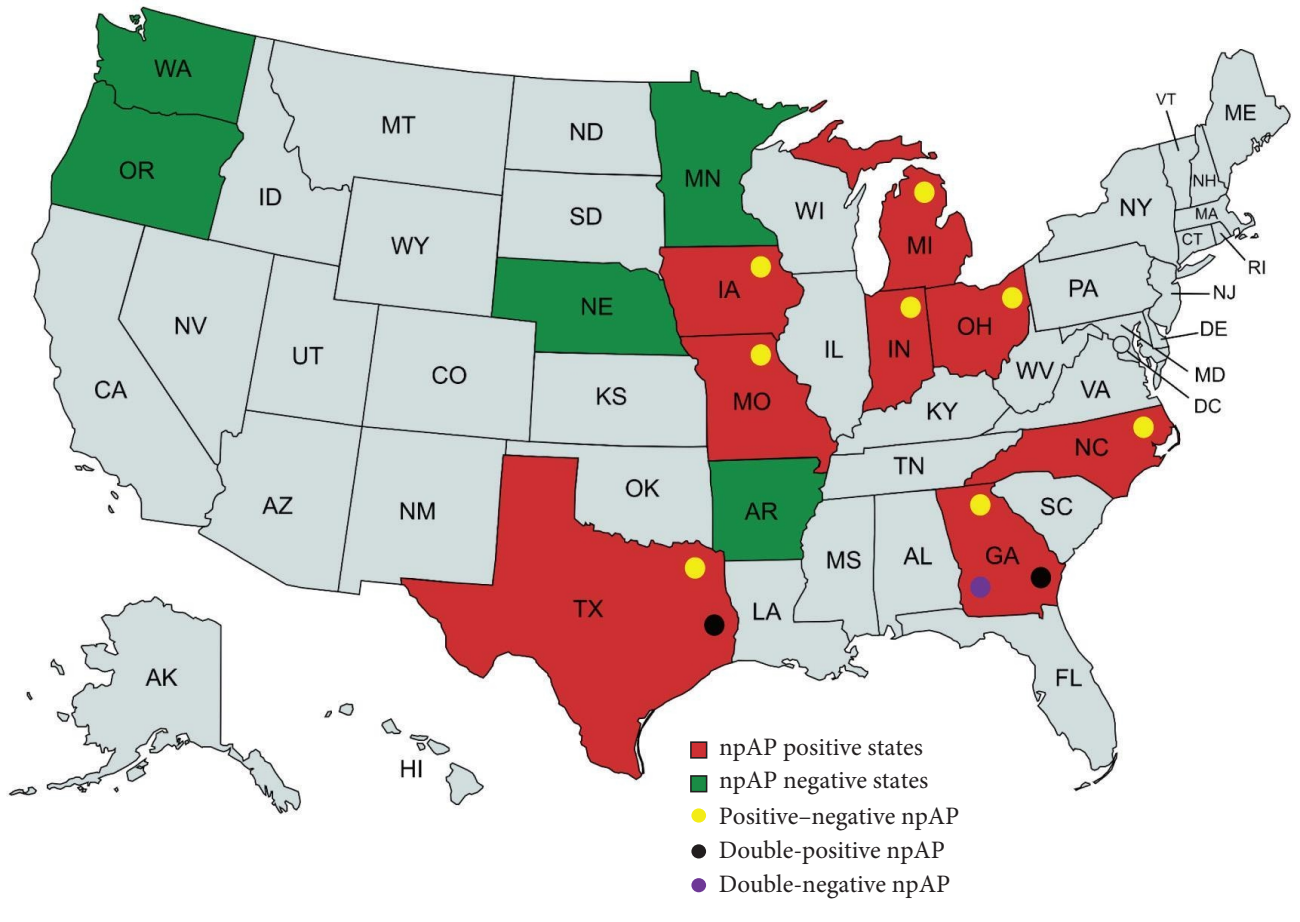
npAP-qPCR positive states in red and negative states in green. The yellow circle represents the positive–negative npAP which was first discovered and thought to be the most widely spread. The positive–negative npAP has a unique insertion in the *HMTp210* gene and lacking the capsular polysaccharide transporter gene, *hctA*. The black circle corresponds to npAP double-positive, which has the same unique insertion in the *HMTp210* gene, but also have the *hctA* gene, while the violet circle denotes the double-negative npAP with a similar unique insertion in the *HMTp210* gene, but with mismatches at the primer and probes annealing sites and lacking *hctA*. (This figure was created by mapchart.net).

The 28 positive sites were subsequently tested by the new differential qPCR assays. Twenty-four sites (85.71%) tested positive by np-*HMTp210* qPCR and negative by *hctA* qPCR (positive–negative sites), confirming that the positive screening qPCR signal was indeed due to the presence of npAP in these 24 NHL sites. However, the remaining four sites (three SFMH layer sites in Georgia and one MAC layer site in Texas) showed positive signals by both *HMTp210* insertion and *hctA* assays (double-positive sites; Table 3 and Figure 3).

### 3.2. Bacterial Isolation and MALDI-TOF

We isolated 17 npAP isolates from six out of the 28 PCR-positive sites: two sites from Ohio, two sites from Georgia, and one site each from North Carolina and Michigan. All isolates tested positive for AP using MALDI-TOF and *recN* qPCR. Results from the two differential qPCR assays showed that the isolates from Ohio, North Carolina, and Michigan were positive for *HMTp210* insertion and negative for *hctA* (positive–negative) which is consistent with their classification as npAP (Table 4). A particularly interesting group of isolates was acquired from two sites in Georgia, which are two out of the four double-positive sites mentioned in Section 3.1. From these two Georgian double positive sites, nine isolates (five from one site and four from the other site) were acquired. Single colonies from each isolate was picked, used to DNA extraction and tested by the two differential qPCR assays. Those isolates were further divided into two distinct npAP populations based on their results on the two differential qPCR assays. Seven out of the nine isolates were positive for both *HMTp210* insertion and *hctA* assays (double-positive isolate; Table 4). Surprisingly, the remaining two isolates (obtained from one site) were negative for both *HMTp210* insertion and *hctA* assays and will be referred to as double-negative isolates (Table 4).

### 3.3. WGS and ANI

The WGS analysis of the isolates confirmed that differential qPCR results were consistent with the presence or absence of the target sequence. The npAP positive–negative isolates (eight out of 17 isolates) exhibited unique lengthy insertions in *HMTp210* and lacked the *hctA* gene in their genomes. Four double-positive isolates and two double-negative isolates were submitted for WGS. The four double-positive isolates demonstrated the presence of the same unique insertions in *HMTp210*, in addition to the presence of the *hctA* gene. The WGS of the double-negative isolates displayed the presence of the lengthy insertions in the *HMTp210* as well, but the sequence of their *HMTp210* insertions mismatched the developed qPCR assay primers and probe in their annealing sites. However, these double negative isolates still lacked the *hctA* gene, similar to the positive–negative isolates. The ANI score of all representative isolates ranged from 96.57% to 96.88%, which is above the cut-off threshold of 96% [26] indicating that indeed all these isolates belong to AP species.

## 4. Discussion

IC is one of the significant challenges facing the poultry industry in the United States. Since the reemergence of the disease in December 2018 in Pennsylvania [9], a series of outbreaks occurred in multiple states [5]. Following the index case in Ohio, active outbreak investigation was carried out in the surrounding flocks in late 2020 and early 2021. During this oubreak investigation effort, it was recognized that completely normal layer flocks, which were not vaccinated nor previously exposed to IC (NHL flocks), showed positive signals by all available IC-specific PCR assays [4, 5, 27]. These results were then potentially explained by the discovery of a nonpathogenic population of AP when AP isolates could be retreived from some of these NHL flocks in 2021 [16].

The previously published genetic characterization of npAP isolates [16] revealed that they are distinct from the pAP in some genetic markers like *HMTp210* gene unique insertions and the lack of capsular polysaccarchride locus (including *hctA*), while their ANI score of approximately 96%, confirms that they are still within the AP species according to the established threshold for species definition [26, 28–30]. Given our limited understanding of this newly discovered npAP population, the extent and the scale of the diagnostci confusion remained unknown. Therefore, this surveillance pilot study was conducted to investigate the prevalence of npAP in the US commercial layer industry.

Using the screening *recN* qPCR assay [5], 28 out of 80 NHL sites tested positive for AP in the current study and were subsequently confirmed to be due to npAP either by the use of two newly developed differential qPCR assays [17] and/or by WGS. Additionally, bacterial isolation from six of these sites (6/28) and WGS was in full agreement with the PCR results and further confirmed the presence of npAP. These results suggest that the preliminary prevalence estimate of npAP in the layer sites tested in our pilot study is 35%. This means that approximately one out of every three NHL commercial sites tested positive by qPCR would be incorrectly diagnosed as positive for IC.

Out of the 13 US states tested, eight were positive for npAP, and from the five negative states (Arkansas, Washington, Oregon, Nebraska, and Minnesota), only a total of 10 sites from all five states were tested, which is a relatively small number. This limited sample size may be the reason these states tested negative for npAP, suggesting that more sites should be tested for a conclusive result. Probably for the same reason, lack of enough samples, apart from Pennsylvania, which was not tested in this study, Arkansas is the only top 10 table egg producing state where npAP was not detected.

More positive sites for npAP were detected in the MAC production housing systems (23/40, 57.5%) compared to other housing systems that use an all-in-all-out strategy, such as SFMH sites (4/24, 16.6%) and SFSH sites (1/16, 6.25%). As documented before, large MAC sites are more likely to experience continuous outbreaks with various infectious agents, including AP, due to the continuous circulation of the viable bacterium between older carrier birds and incoming susceptible pullets, which helps maintain the bacterium in the environment [31–33]. This could explain why we reported a higher incidence of npAP in MAC sites. Consequently, the explanation for this lower prevalence in all-in-all-out housing systems could be that even if these flocks were exposed to npAP in previous cycles, they could have cleared it with depopulation and our sampling timing may not have aligned with their exposure. For this reason, it is critical to include MAC in AP surveillance studies, as the bacterium is likely to persist longer in these systems, increasing the likelihood of detecting true positive signals.

A wide age range of laying hens, from 17 to 106 wks., was included in the study and npAP detection was not restricted to a specific age range, as it was found across the entire range. This suggests that all chickens from the onset to the end of lay are susceptible to npAP, which is similar to pAP [34]. However, the age averages was significantly higher (*p*=0.0039) in the positive sites compared to the negative ones, showing that the newly discovered npAP is preferentially associated with older mature hens (specifically older than 49 wks), also similar in that respect to the pAP. Regarding the *C*_T_ values across positive sites in the tested states, there was no significant difference observed, with average *C*_T_ values being high at around 29 ± 2.62. This finding contrasts with what we typically observe in acute IC cases, which usually have lower *C*_T_ values (<25). The higher CT values associated with npAP cases could potentially be explained by chronic or low replication levels of npAP strains.

In this study, we observed variability in the npAP population, including positive–negative, double-positive, and double-negative npAP strains. Initially, the double-positive sites were thought to potentially result from a mixed infection of npAP and pAP. However, WGS analysis of individual colonies revealed that the double-positive isolates are npAP isolates possessing the capsular polysaccharide locus, including the *hctA* export gene. In contrast, both the positive–negative and double-negative npAP strains lack *hctA*, leading to the absence of the bacterial capsule. A common and unique feature across all npAP strains is the presence of lengthy insertions in the *HMTp210* gene. These findings underscore the need to further investigate and identify the primary virulence factors of AP. Previous research has established that the capsular polysaccharide is a key virulence factor, as nonencapsulated strains either fail to cause clinical symptoms [35] or display reduced virulence [36]. However, our results revealed a double-positive npAP population with both the capsule and unique insertions in the *HMTp210* gene. This suggests that, at this time *HMTp210* remains the only virulence factor, that is consistently different between these two bacterial populations, with its function potentially disrupted by these insertions in the npAP strains. However, a more comprehensive genomic comparison studies are required to better identify and study the presence of absence of other pathogenicity factors in the two bacterial populations. Additionally, the classification of the three described npAP population, positive–negative, double-positive, and double-negative, is based on the differential qPCR results, confirmed by WGS. However, confirming the pathogenicity, or lack thereof, in all three population requires in vivo challenge studies in susceptible chickens. Recently, our group conducted a challenge study using infraorbital sinus injection in 26-week-old naïve layers, including the three distinct classified npAP isolates. Results from the challenge study showed no significant differences between the npAP-challenged groups and the negative control group in terms of clinical scores, gross lesion scores, or histopathological lesions, while the positive control group was significantly higher than all other groups. The results of this challenge study support the assumption that isolates from the current surveillance study are npAP (unpublished data).

From a diagnostic perspective, these results emphasize the importance of using the *recN* screening assay [5] as the first line of detection, as it successfully identified all npAP strains. However, this assay cannot differentiate between pAP and npAP. It is crucial to have a robust differential diagnostic tool, preferably a qPCR assay, which can distinguish between all npAP populations and the pAP. Although the newly developed differential qPCR assays [17] work well to confirm most npAP cases (24 out of 28, representing 85.71%), some modifications are required to make this assay universally reliable, which would be a useful diagnostic tool that can differentiate between pAP and npAP in all cases.

Mixed infections of different npAP populations were demonstrated by isolating double-positive and double-negative isolates from the same layer flock. However, it remains a question whether mixed infections of pAP and npAP can occur in the same flock. While we did not confirm any such mixed infections in the tested commercial layer flocks during our study, we did identify such infection in one clinical backyard flock that was not part of the surveillance study. The role of backyard birds in npAP transmission is still unknown, but they could potentially act as a source of bacterial dissemination to surrounding NHL flocks, similar to their documented role as a reservoir for the pAP [33]. In this study, we chose to focus on the commercial layer industry, as they appeared to be the industry most vulnerable to the effects of this diagnostic confusion in the face of an expanding outbreak. However, the extent of this problem in the backyard flocks as well as the commercial broiler industry remains unclear.

## 5. Conclusions

In conclusion, multiple npAP populations are circulating in the US layer industry, with the results from this pilot study suggesting high prevalence of approximately 35%. This high prevalence rate complicates the diagnostic landscape, as current diagnostic tools, including qPCR, bacterial isolation, and MALDI-TOF, cannot differentiate between npAP and pAP. The recently developed differential qPCR assays addressed the diagnostic confusion in 85.71% of cases; however, further modifications are necessary for them to be universally adopted as robust differential tools. Consequently, no practical diagnostic test can be relied upon as confirmatory tests for IC diagnosis, particularly in flocks with no active clinical signs. The only confirmatory diagnostic evidence can be achieved by bacterial isolation combined with WGS generation and analysis. However, this is not a practical diagnostic strategy, especially in cases when surveillance or outbreak investigation needs to be conducted in NHL chicken flocks, in the face of a rapidly expanding outbreak. Additionally, further studies are required to investigate the pathogenicity or lack thereof, and the potential immunogenicity, or lack thereof, of these newly discovered npAP isolates via chicken challenge model studies. Furthermore, comprehensive genomic comparisons for a better understanding of the genus *Avibacterium* and the other species contained in that genus remain crucially needed.

## Figures and Tables

**Table 1:** Description of tested samples among 13 US states.

State	Number of samples	Number of birds	Age (weeks)			Type and number of sampled facility			Date range
			Range	Average	SD	MAC	SFMH	SFSH	
Arkansas	30	150	24–41	33.6	6.58	−	−	5	December 2022–March 2023
Georgia	60	300	61–92	73.77	8.58	−	10	−	October 2023
Indiana	48	240	76–98	85.25	6.9	7	−	1	October 2023–December 2023
Iowa	90	450	38–104	81.33	14.8	8	−	1	April 2023–July 2023
Michigan	27	135	27–88	62	19.81	3	−	1	July 2023–March 2024
Minnesota	6	30	61	61	0	−	1	−	October 2023
Missouri	30	150	40–85	60.6	18	1	−	1	December 2022 and April 2023
Nebraska	12	60	40–71	59.28	11.75	2	−	−	July 2023
North Carolina	162	810	23–82	46.66	20.63	−	10	2	July 2023–January 2024
Oregon	12	60	68–75	71.5	4.94	1	−	−	April 2023
Ohio	162	810	17–97	63.89	23.03	7	3	5	December 2022–June 2023
Texas	59	295	54–106	91.7	17.42	10	−	−	September–October 2023
Washington	12	60	76–95	85.5	13.43	1	−	−	April 2023

*Note:* Number of samples refers to the number of oropharyngeal swab pools (five swabs per pool), while the number of birds denotes the total number of individual birds swabbed before pooling. − denotes no sample collected.

Abbreviations: MAC, multiage layer complex; SD, standard deviation; SFMH, single-flock-multihouse; SFSH, single-flock-single-house.

**Table 2:** Primer and probe sequences for *recN*, *np*-*HMTp210*, *and hctA* qPCR *assays*.

Name	Sequence (5ˊ–3ˊ)	Reference
*recN* forward primer	GAACAAGACCCTTATCGCTTACAAG	[5]
*recN* reverse primer	ACTCACTAATTCTTCCGCTTTTACATT	
*recN* probe	FAM/CAGGCACTGCAATTAGCCCGCAA/BHQ-1	

*np-HMTp210* forward primer	AGAAAACAGCAATCCGACTG	[17]
*np-HMTp210* reverse primer	GTTAAGCTATCCTGAACTGC	
*np-HMTp210* probe	56FAM/AGAGAAACT/ZEN/GGAACAAGCGGCTAAAGATGG/3IABkFQ	
*hctA* forward primer	ATTGTCTAATAGCGGTTGGA	
*hctA* reverse primer	ATTGATGAAGTGATTGCCGTT	
*hctA* probe	56-FAM/AGAAACTAA/ZEN/AATAATGGAACGATCTTTGCG/3IABkFQ	

**Table 3:** Summary of *recN* as well as the differential qPCR results for all tested naïve-healthy layer sites per US state.

State	Number of *recN* positive/total samples	Number of *recN* positive/total of each housing type			Number of *recN* positive /total sites	Age range by weeks of positive flocks	Differential qPCR results	
		MAC	SFMH	SFSH			np-*HMTp210*	*hctA*
Arkansas	0/30	−	−	0/5	0/5	NA	NT	NT

Georgia	18/60 (30%)	−	3/10	−	3/10 (30%)	70–74	+ve	+ve

Indiana	11/48 (22.91%)	2/7	−	0/1	2/8 (25%)	87–98	+ve	–ve

Iowa	24/90 (26.66%)	2/8	−	0/1	2/9 (22.22%)	74–104	+ve	–ve

Michigan	13/27 (48.14%)	2/3	−	1/1	3/4 (75%)	27–84	+ve	–ve

Minnesota	0/6	−	0/1	−	0/1	NA	NT	NT

Missouri	22/30 (73.33%)	1/1	−	0/1	1/2 (50%)	40–85	+ve	–ve

Nebraska	0/12	0/2	−	−	0/2	NA	NT	NT

North Carolina	9/162 (5.55%)	−	1/10	0/2	1/12 (8.33%)	87–98	+ve	–ve

Oregon	0/12	0/1	−	−	0/1	NA	NT	NT

Ohio	90/162 (55.55%)	6/7	0/3	0/5	6/15 (40%)	17–97	+ve	–ve

Texas	44/59 (74.57%)	10/10	−	−	10/10 (100%)	54–106	(10/10 +ve)	(1/10 +ve)

Washington	0/12	0/1	−	−	0/1	NA	NT	NT

Total positive	231/710 (32.53%)	23/40 (57.5%)	4/24 (16.6%)	1/16 (6.25%)	28/80 (35%)	17–106	—	—

*Note:* Samples refers to oropharyngeal swab pools. − denotes no sample collected. +ve, positive; –ve, negative.

Abbreviations: MAC, multiage layer complex; NA, nonapplicable; NT, not tested; SFMH, single-flock-multihouse; SFSH, single-flock-single-house.

**Table 4:** Descriptive summary of the three npAP populations and their corresponding qPCR results.

npAP population	Positive samples/total positives	Positive isolates/total positives	Capsular polysaccharide locus (including *hctA*)	Lengthy *HMTp210* insertions	np-*HMTp210* primers and probe	*recN* screening qPCR assay	np-*HMTp210* qPCR assay	*hctA* qPCR assay
Positive–negative npAP	85.7% (24/28 positive sites)	47.0% (8/17 isolates)	Absent	Present	Matches	+ve	+ve	−ve
Double-positive npAP	14.3% (4/28 positive sites)	41.2% (7/17 isolates)	Present	Present	Matches	+ve	+ve	+ve
Double negative npAP	Not detected at the site level Only detected as isolates	11.8% (2/17 isolates)	Absent	Present	Mismatches	+ve	−ve	−ve

*Note:* Samples refers to oropharyngeal swab pools. “−ve” denotes negative, “+ve” denotes positive, *recN* qPCR assay is a screening AP specific assay that cannot differentiate between pAP and npAP. np-*HMTp210* (targets a unique insertion in the *HMTp210* of npAP) and *hctA* (targets a capsular transport gene) are the recently developed differential qPCR assays. Positive–negative npAP refers to npAP that tested positive with the np-*HMTp210* assay and negative with the *hctA* assay. Double-positive npAP tested positive on both the np-*HMTp210* and *hctA* assays, while double-negative npAP were negative on both np-*HMTp210* and *hctA* assays.

## Data Availability

The complete and whole genome sequences of the 11 npAP isolates are available in the GenBank database under the following accession numbers: CP173231–CP173234, JBJBHF000000000 (positive–negative npAP), CP173229, CP173230, CP173236, JBJBHD000000000 (double-positive npAP), and CP173228, JBJBHC000000000 (double-negative npAP).

## Nomenclature

ANI: Average nucleotide identity
AP: *Avibacterium paragallinarum*
IC: Infectious coryza
MAC: Multiage layer complexes
MALDI-TOF: Matrix-assisted laser desorption ionization time-of-flight
npAP: Nonpathogenic AP
OP: Oropharyngeal
pAP: Pathogenic AP
qPCR: Quantitative real-time PCR
SFMH: Single-flock-multihouse
SFSH: Single-flock-single-house
XIPC: Internal positive control.

## Supporting Information

Supporting Information It contains Table S1 and its caption is detailed description of all samples collected and tested in this study. State, site housing system, house names, age, history of the flock, and the results of each tested swab pool on the *recN* screening qPCR and the type of npAP based on the results of the two newly developed differential qPCR assays.

## References

[1] Blackall P. J. (1999). Infectious Coryza: Overview of the Disease and New Diagnostic Options. *Clinical Microbiology Reviews*.

[2] Blackall P. J., Christensen H., Beckenham T., Blackall L. L., Bisgaard M. (2005). Reclassification of *Pasteurella gallinarum*, [Haemophilus] paragallinarum, *Pasteurella avium* and *Pasteurella volantium* as *Avibacterium gallinarum* gen. nov., comb. nov., *Avibacterium paragallinarum* comb. nov., *Avibacterium avium* comb. nov. and *Avibacterium volantium* comb. nov. *International Journal of Systematic and Evolutionary Microbiology*.

[3] Byarugaba D. K., Minga U. M., Gwakisa P. S., Katunguka-Rwakishaya E., Bisgaard M., Olsen J. E. (2007). Investigations of the Occurrence of *Avibacterium paragallinarum* Infections in Uganda. *Avian Diseases*.

[4] Corney B., Diallo I., Wright L. (2008). Rapid and Sensitive Detection of *Avibacterium paragallinarum* in the Presence of Other Bacteria Using a 5′ Taq Nuclease Assay: A New Tool for Diagnosing Infectious Coryza. *Avian Pathology*.

[5] Kuchipudi S. V., Yon M., Surendran Nair M. (2021). A Highly Sensitive and Specific Probe-Based Real-Time PCR for the Detection of *Avibacterium paragallinarum* in Clinical Samples From Poultry. *Frontiers in Veterinary Science*.

[6] Wen S., Chen X., Xu F., Sun H., Reddy H. (2016). Validation of Reference Genes for Real-Time Quantitative PCR (qPCR) Analysis of *Avibacterium paragallinarum*. *PloS One*.

[7] Krylova E., Bogomazova A., Kirsanova N. (2023). Development and Validation of PCR Diagnostic Assays for Detection of *Avibacterium paragallinarum* and *Ornithobacterium rhinotracheale*. *Veterinary Sciences*.

[8] Blackall P. J., Soriano-Vargas E. (2020). Infectious Coryza and Related Bacterial Infections. *Diseases of Poultry*.

[9] Sandoval V. E., Terzolo H. R., Blackall P. J. (1994). Complicated Infectious Coryza Outbreaks in Argentina. *Avian Diseases*.

[10] Yamamoto R., Hofstad M. S., Calnek B. W., Helmboldt C. F., Reid W. M., Yoder H. W. (1972). Infectious Coryza. *Diseases of Poultry*.

[11] Yamamoto R., Hofstad M. S., Calnek B. W., C. F. Hembolt Reid, Yoder W. M. (1978). Infectious Coryza. *Diseases of Poultry*.

[12] Dunn P. A., Wallner-Pendleton E. A., Love B. C., Kahn M., Opitz M. Infectious Coryza Update.

[13] Davison S., Tracy L., Kelly D. J. (2024). Infectious Coryza in Pennsylvania. *Avian Diseases*.

[14] Byukusenge M., Nissly R. H., Li L. (2020). Complete Genome Sequences of Seven *Avibacterium paragallinarum* Isolates From Poultry Farms in Pennsylvania, USA. *Microbiology Resource Announcements*.

[15] Gingerich E. Layer Health Report for October 1, 2022 to October 1, 2023.

[16] Hashish A., Chaves M., Macedo N. R. (2023). Complete Genome Sequences Generated Using Hybrid Nanopore-Illumina Assembly of Two Non-Typical *Avibacterium paragallinarum* Strains Isolated from Clinically Normal Chicken Flocks. *Microbiology Resource Announcements*.

[17] Shelkamy M. M. S., Hashish A., Chaves M. (2024). Development and Validation of PCR Assays for Improved Diagnosis of Infectious Coryza by Differentiating Pathogenic and Nonpathogenic *Avibacterium paragallinarum*. *Avian Diseases*.

[18] Browne R. H. (1995). On the use of a Pilot Sample for Sample Size Determination. *Statistics in Medicine*.

[19] Julious S. A. (2005). Sample Size of 12 per Group Rule of Thumb for a Pilot Study. *Pharmaceutical Statistics: The Journal of Applied Statistics in the Pharmaceutical Industry*.

[20] National Agricultural Statistics Service (NASS) (2023). Chickens and Eggs.

[21] Cameron A. R., Baldock F. C. (1998). A New Probability Formula for Surveys to Substantiate Freedom From Disease. *Preventive Veterinary Medicine*.

[22] Fosgate G. T. (2009). Practical Sample Size Calculations for Surveillance and Diagnostic Investigations. *Journal of Veterinary Diagnostic Investigation*.

[23] Houston D. D., Azeem S., Lundy C. W. (2017). Evaluating the Role of Wild Songbirds or Rodents in Spreading Avian Influenza Virus Across an Agricultural Landscape. *PeerJ*.

[24] Holland R., Wilkes J., Rafii F. (1996). Rapid Identification of Intact Whole Bacteria Based on Spectral Patterns Using Matrix-assisted Laser Desorption/Ionization With Time-of-flight Mass Spectrometry. *Rapid Communications in Mass Spectrometry*.

[25] Richter M., Rosselló-Móra R., Oliver Glöckner F., Peplies J. (2016). JSpeciesWS: A Web Server for Prokaryotic Species Circumscription Based on Pairwise Genome Comparison. *Bioinformatics*.

[26] Viver T., Conrad R. E., Rodriguez-R L. M. (2024). Towards Estimating the Number of Strains that Make up a Natural Bacterial Population. *Nature Communications*.

[27] Chen X., Miflin J. K., Zhang P., Blackall P. J. (1996). Development and Application of DNA Probes and PCR Tests for Haemophilus Paragallinarum. *Avian Diseases*.

[28] Konstantinidis K. T., Tiedje J. M. (2005). Genomic Insights That Advance the Species Definition for Prokaryotes. *Proceedings of the National Academy of Sciences of the United States of America*.

[29] Richter M., Rosselló-Móra R. (2009). Shifting the Genomic Gold Standard for the Prokaryotic Species Definition. *Proceedings of the National Academy of Sciences of the United States of America*.

[30] Rodriguez-R L. M., Jain C., Conrad R. E., Aluru S., Konstantinidis K. T. (2021). Reply to: “Re-Evaluating the Evidence for a Universal Genetic Boundary Among Microbial Species". *Nature Communications*.

[31] Halvorson D. A. (2011). Biosecurity on a Multiple-Age Egg Production Complex: A 15-Year Experience. *Avian Diseases*.

[32] Kreager K., Shane S. M. (1995). Principles of Disease Prevention in Commercial Layers. *Biosecurity in the Poultry Industry*.

[33] Crispo M., Blackall P., Khan A. (2019). Characterization of an Outbreak of Infectious Coryza (*Avibacterium paragallinarum*) in Commercial Chickens in Central California. *Avian Diseases*.

[34] Yamamoto R., Barnes H. J., Calnek B. W., Beard C. W., Reid W. M., Yoder H. W. (1991). Infectious Coryza. *Diseases of Poultry*.

[35] Sawata A., Nakai T., Kume K., Yoshikawa H., Yoshikawa T. (1985). Lesions Induced in the Respiratory Tract of Chickens by Encapsulated or Nonencapsulated Variants of Haemophilus Paragallinarum. *American Journal of Veterinary Research*.

[36] Tu T.-Y., Hsieh M.-K., Tan D.-H. (2015). Loss of the Capsule Increases the Adherence Activity But Decreases the Virulence of *Avibacterium paragallinarum*. *Avian Diseases*.

